# Overcoming isolation barriers: *in vitro* cultivation and molecular identification of *Cephaleuros virescens* associated with African mahogany

**DOI:** 10.3389/fpls.2026.1794889

**Published:** 2026-03-13

**Authors:** Fabíola Teodoro Pereira, Natália Cassia de Faria Ferreira, Ângelo Márcio da Silva Fuzzo, Thiago Alves Santos de Oliveira, Elizabeth Amélia Alves Duarte, Daniel Diego Costa Carvalho

**Affiliations:** 1 Phytopathology Laboratory, State University of Goiás (UEG), Ipameri, Brazil; 2 Multidisciplinary Center of Barra, Federal University of Western Bahia (UFOB), Bahia, Brazil; 3 Afya Faculty of Medical Sciences, Cruzeiro do Sul, Brazil

**Keywords:** 18S rRNA, algal spot, forest pathology, *Khaya ivorensis*, phycology

## Abstract

Algae of the genus *Cephaleuros* are specialized plant pathogens prevalent in tropical ecosystems; however, *in vitro* studies remain constrained by the historical difficulty of establishing axenic cultures. This study aimed to isolate, identify, and establish a functional cultivation protocol for *Cephaleuros* associated with algal leaf spot on African mahogany (*Khaya ivorensis*) in the Brazilian Cerrado. Isolation was successfully achieved through a phased liquid-to-solid transition: symptomatic leaf fragments were initially cultivated in liquid Trebouxia medium under constant agitation (120 rpm) for 3 days, followed by the subculturing of algal mycelium onto Trebouxia agarized medium to obtain pure colonies of isolate H-27-03. Direct isolation on solid media failed across all tested substrates (PDA, PSA, BBM, and HLE), confirming the requirement for a liquid phase to facilitate sporangia release. Surface disinfestation of host tissue proved critical, significantly enhancing algal development (yielding a growth score of Note 2) compared to non-disinfested fragments (Note 1). Phylogenetic analysis of the 18S rRNA gene sequence (GenBank MH042530) confirmed the species as *Cephaleuros virescens* with >98% identity. This study represents the first report of *C. virescens* causing algal leaf spot on *K. ivorensis* in the Brazilian Cerrado. By overcoming isolation barriers through standardized nutritional and physical parameters, this work provides a fundamental framework for future research on the epidemiology, physiology, and management of this emerging forest pathogen.

## 1 Introduction

African mahogany (*Khaya ivorensis* A. Chev) has emerged as a forest species of significant economic importance in Brazil, increasingly adapted to local environmental conditions through rigorous selection processes (Hassler, 2018; Faria et al., 2024). Because of the high added value of its wood in both national and international markets, particularly for premium furniture manufacturing, large-scale cultivation of *K. ivorensis* has expanded rapidly (Ferraz Filho et al., 2021). However, the intensification of mahogany plantations, especially within the Brazilian Cerrado, has been accompanied by various phytosanitary challenges, including fungal diseases, as well as infections caused by parasitic green algae of the genus *Cephaleuros* (Castro et al., 2022).

The genus *Cephaleuros* (Trentepohliaceae, Trentepohliales) comprises 29 described species of filamentous green algae that act as specialized parasites of higher plants across a wide host range (Sunpapao et al., 2016). These pathogens typically develop on tree bark, stems, fruits, and leaves, causing the disease clinically known as algal leaf spot (López-Bautista et al., 2002; Nelson, 2008). Characterized by branched filaments that form irregular, protruding colonies with a distinct orange or ferruginous coloration, *Cephaleuros* spp. can lead to extensive defoliation, tissue necrosis, and reduced plant vigor (Pereira et al., 2020). The development of these algae, particularly pathogenic species such as *C. virescens* and *C. parasiticus*, is highly dependent on tropical and subtropical conditions of high temperature and humidity, as well as the physiological and nutritional status of the host (López-Bautista et al., 2002; Brooks et al., 2015).

Despite the increasing taxonomic and molecular interest in the order Trentepohliales due to its prevalence in tropical ecosystems, scientific progress remains severely hindered (Zhu et al., 2015; Borgato et al., 2022). A primary obstacle is the historical difficulty of establishing axenic cultures in artificial media (Vasconcelos et al., 2018). Furthermore, the frequent occurrence of lichenized species within this group often leads to significant interpretive errors in classical taxonomy (Borgato et al., 2022). Consequently, the lack of standardized *in vitro* cultivation protocols has limited deeper investigations into the physiology, pathogenicity, and control strategies for these unique phytopathogens.

In the Brazilian Cerrado, the recent emergence of characteristic algal spot lesions on *K. ivorensis* has occurred without precise etiological identification, representing a critical gap in forest pathology (Pereira et al., 2020). Establishing an axenic culture is the fundamental prerequisite for understanding physiology and developing management strategies for this pathogen in mahogany plantations. Therefore, the objective of this study was to isolate, identify through molecular phylogeny (18S rRNA), and establish an effective *in vitro* cultivation protocol for the algae species causing leaf spot on African mahogany in the Brazilian Cerrado. This work provides a novel approach to the isolation of this microorganism and expands the molecular understanding of *C. virescens*.

## 2 Materials and methods

### 2.1 Isolation in liquid media

*K. ivorensis* leaf exhibiting symptoms of algal leaf spot were collected from a reforestation area located at Catalão City, Goiás State, Brazil (18°09′S, 47°54′N, 860 m). Small fragments (5 × 5 mm) comprising the transition area between the injured tissue and healthy tissue were excised and then disinfected in a series, as follows: 70% alcohol (30 s), 1% sodium hypochlorite (30 s), and SDW (3 × 60 s).

The disinfected (D) and nondisinfected (ND) fragments were sown in Petri dishes (1 fragment dish^−1^) containing culture media: (a) potato dextrose agar (PDA; containing g L^−1^: 200 g potato, 20 g dextrose, and 20 g agar); (b) potato sucrose agar (PSA, containing g L^−1^: 200 g potato, sucrose 20 g, and agar 20 g); (c) bold basic medium [BBM; containing g L^−1^: (1) 25 g NaNO_3_, (2) 2.5 g CaCl_2_·2H_2_O, (3) 7.5 g MgSO_4_·7H_2_O, (4) 7.5 g K_2_HPO_4_, (5) 17.5 g KH_2_PO_4_ (6) 2.5 g NaCl, (7) 50.0 g EDTA, (8) 31.0 g KOH, (9) 4.98 g FeSO_4_·7H_2_O, (10) 11.42 g H_3_BO_3_, (11) 1 mL H_2_SO_4_, (12) micronutrient solution containing g L^−1^: 8.82 g ZnSO_4_·7H_2_O, 1.44 g MnCl_2_·4H_2_O, 0.71 g MoO_3_, 1.57 g CuSO_4_·5H_2_O, 0.49 g Co(NO_3_)2.6H_2_O; prepared with 10 mL of solutions 1–6, 940 mL of SDW, 1 mL of solutions 7–12, and 22 g agar]; (d) agarized host leaf extract (HLE; containing 1 L of filtrate from 100 g of the crushed healthy mahogany leaves, 20 g sucrose, and 20 g agar); and (e) Trebouxia (g L^−1^: containing 10 g proteose peptone, 20 g glucose, 60 mL Bristol solution, and 20 g agar). Bristol solution was prepared (containing g L^−1^) as follows: 0.097 g FeCl_2_, 0.004 g MnCl_2_, 0.005 g ZnCl_2_, 0.002 g CaCl_2_, 0.004 g Na_2_MoO_4_, and 20 g agar). After inoculation in liquid media (5 fragments Erlenmeyer^−1^), the flasks were put into the shaker at 120 rpm at 25 °C and cool-white fluorescent lamps (400–700 nm) for 3 days, which is the isolation protocol as per previous evidence that mechanical movement facilitates sporangia release in Trentepohliaceae (Vasconcelos et al., 2018). After growing, algal mycelium fragments were transferred from the Trebouxia liquid medium to Trebouxia agarized media. This procedure allowed us to obtain the *C. virescens* isolate H-27-03.

The growth evaluation was carried out according to the rating scale adapted from Ponmurugan et al. (2010) as follows: Note 0—absence of growth; Note 1—Algae colonize 1% to 16% of the surface area (agar medium) or up to 16% of the volume (liquid medium) of the culture medium; Note 2—Algae colonize 16.1% to 33% of the surface area (agar medium) or 16.1% to 33% of the volume (liquid medium) of the culture medium; Note 3—Algae colonize 33.1% to 50% of the surface area (agar medium) or 33.1% to 50% of the volume (liquid medium) of the culture medium; and Note 4—Algae colonize more than 50.1% of the surface area (agar medium) or more than 50.1% of the volume (liquid medium) of the culture medium. Statistical analyses were performed on data from liquid media isolation. Differences between culture media were analyzed using the Kruskal–Wallis test, while the effect of surface disinfestation (D *vs*. ND) within the successful medium (Trebouxia) was evaluated using the Mann–Whitney test (*p* < 0.05). The experiment was conducted in a randomized design, with five replicates (Erlenmeyer flasks) per treatment (culture medium x asepsis), totaling 25 fragments and 5 Erlenmeyer analyzed per condition.

The ordinal scale was adapted to quantify algal development where traditional radial growth measurements are hindered by the slow-growing and tufted nature of *Cephaleuros* colonies. To ensure scoring consistency and eliminate subjectivity, evaluations were performed independently by two experienced researchers using standardized visual reference templates, followed by a consensus review.

### 2.2 Molecular and phylogenetic analyses

The DNA was extracted from pure colonies’ culture of isolate H-27-03, using the UltraClean^®^ Microbial DNA Isolation kit (MoBio, USA), following the manufacturer’s recommendations. DNA integrity and quantity were verified by electrophoresis in 1.0% agarose gel stained with ethidium bromide and viewed under ultraviolet light, followed by photodocumentation and e Qubit^®^ 2.0 Fluorometer (Invitrogen), respectively.

Sequences for the small subunit rRNA gene (18S rDNA) were amplified with the primers 18SHf (GCCCTTCCGTCAATTCCTTTAAGTTTCAGC) and 18SLr (CACCTACGGAAACCTTGTTACGACTT) described by Hamby et al. (1988). Reactions were prepared with the following reagents and concentrations: 60 g of DNA from each sample; 1× of Taq DNA polymerase buffer; 3.7 mM of MgCl_2_; 0.6 pmol/μL of dNTPs; 0.4 pmol/μL of each primer; 5 U of Taq DNA polymerase (Invitrogen), with final volume adjusted to 50 μL with ultrapure water. Amplification cycles were performed in the Veriti Thermal Cycler PCR (Applied Biosystems) with an initial denaturation temperature of 95 °C for 3 min; 30 amplification cycles at 95 °C for 1 min; 52 °C for 1 min; 72 °C for 2 min and a final extension temperature of 72 °C for 10 min. The amplified products were viewed in 1% agarose gel, stained with ethidium bromide and visualized under ultraviolet light. Then, the amplicons were purified using the PureLink™ PCR Purification kit (Invitrogen), quantified with a Qubit^®^ 2.0 Fluorometer (Invitrogen) for subsequent nucleotide identification, using the automatic sequencer *ABI-Prism 3500 Genetic Analyzer* (Applied Biosystems). The sequences were edited and assembled using the program Sequencher 4.1.4 (Gene Code Corporation). The taxonomic identification of the isolates was verified through the database GenBank, using the Basic Local Alignment Search Tool (BLASTn) of the NCBI (http://www.ncbi.nlm.nih.gov).

The sequences generated in this work were combined with the partial 18S rRNA gene sequence of the specimens of *Cephaleuros* (for this genus, there are no type species). The sequence of *Bryopsis plumose* (GenBank accession no. FJ 432630.1), which belongs to the same class (Ulvophyceae) as *Cephaleuros*, was used as an outgroup. The respective accession numbers (GenBank) of the sequences included in the study are presented in the item result (phylogeny). In addition, we compared the names of the green algae available in GenBank with the names adopted from Index Nominum Algarum (http://ucjeps.berkeley.edu/INA.html) and AlgaeBase (http://www.algaebase.org/) to elucidate possible updates of genera, species, and possible synonyms.

The datasets were aligned using Clustal W (Thompson et al., 1994), manually verified using MEGA v.7 (Kumar et al., 2016). The best-fit model of nucleotide evolution to the datasets was selected by both BIC (Bayesian Information Criterion) and AICc (corrected Akaike’s Information Criterion) using jModelTest2 v.1.7 (Guindon and Gascuel, 2003; Darriba et al., 2012). All the four main methods of phylogenetic analysis, Distance (D), Maximum Parsimony (MP), Maximum Likelihood (ML), and Bayesian (B), were used to evaluate the dataset. Phylogenetic analyses were performed in PAUP 4.0b10 (Swofford, 2002) and Mr. Bayes 3.2 (Ronquist and Huelsenbeck, 2003). Bayesian Inference (BI) phylogenetic analysis was also applied to the datasets.

## 3 Results

### 3.1 Isolation in liquid media

To determine the most suitable conditions for the isolation and *in vitro* establishment of *C. virescens*, a qualitative screening was performed using five different culture media: PDA, PSA, BBM, HLE, and Trebouxia (**Table 1**). The evaluation focused on the success or failure of primary isolation from symptomatic leaf fragments (injured tissue) and the subsequent viability of the obtained mycelium. None of the solid culture media evaluated enabled the direct isolation of the alga from host plant tissue fragments exhibiting disease symptoms, regardless of whether the tissues were surface-disinfested or not.

**Table 1:** Growth scores of *Cephaleuros virescens* isolate H-27–03 in different culture media and disinfestation conditions^(1)^.

Culture media	Tissue disinfestation	Liquid medium from injured tissue (Notes 0–4)	Solid medium from injured tissue (Note 0-4)^(2)^	Solid medium from mycelium (Notes 0–4)
PDA	D/ND	0 ± 0 c	0 ± 0	ne
PSA	D/ND	0 ± 0 c	0 ± 0	ne
BBM	D/ND	0 ± 0 c	0 ± 0	ne
HLE	D/ND	0 ± 0 c	0 ± 0	ne
Trebouxia	D	2.0 ± 0.0 a	0 ± 0	4.0 ± 0.0
Trebouxia	ND	1.0 ± 0.0 b	0 ± 0	ne

(D) Surface disinfested; (ND) Non-disinfested; (ne) Not evaluated. ^(1)^Values represent mean ± standard error (*n* = 5). Means followed by different letters in the column “Liquid medium from injured tissue” differ significantly by Kruskal–Wallis test (*p* < 0.001) for media comparison and Mann–Whitney test (*p* < 0.01) for disinfestation effects. Growth scores: (0) no growth; (1) initial filament emergence, algae colonize up to 16% of the volume of the culture medium; (2) evident colonial growth, algae colonize 16.1 to 33% of the volume of the culture medium; (3) full mycelial expansion and purification, algae colonize more than 50.1% of the surface area (agar medium). ^(2)^Statistical analysis for solid media was not performed due to the absolute lack of growth (variance = 0), representing a total biological barrier under the tested conditions.

**Figure 1:**
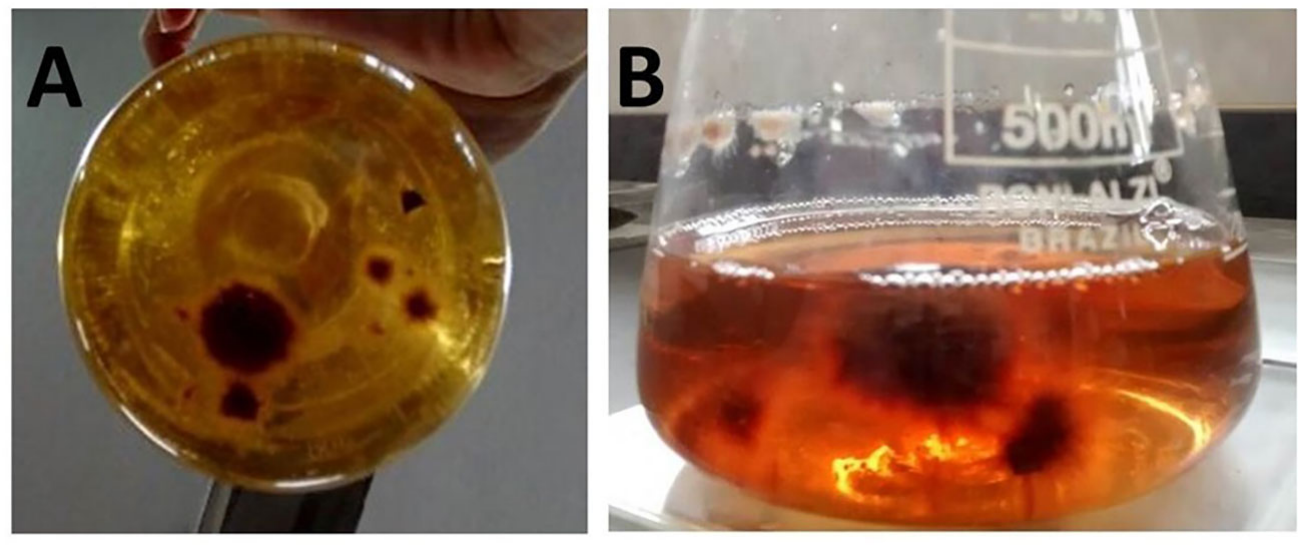
Growth of *Cephaleuros virescens* in non-agarized Trebouxia medium: **(A, B)** colonies of the algae in liquid medium from pieces of disinfected damaged plant tissue, exhibiting a score of 2 on the evaluation scale adapted from Ponmurugan et al. (2010).

The success of *C. virescens* isolation was strictly dependent on the culture medium and the physical state of the substrate (**Figure 1**). The Trebouxia liquid medium was the only one that allowed for the successful establishment of the algal isolate H-27-03. Statistical analysis revealed a significant difference in growth performance between the tested media (Kruskal–Wallis, *H* = 14.0, *p* < 0.001), with the Trebouxia medium being statistically superior to the HLE and other tested formulations. Surface disinfestation of host tissue was a critical factor for isolation efficiency. Within the Trebouxia liquid medium, disinfested fragments (D) showed significantly higher growth scores (2.0 ± 0.0) compared to non-disinfested fragments (ND) (1.0 ± 0.0) (Mann–Whitney, *U* = 25.0, *p* < 0.01). Direct isolation attempts on solid media (PDA, PSA, BBM, and HLE) failed in all instances, resulting in no development or oxidation of the inoculum (Note 0), confirming the requirement for a liquid phase to overcome isolation barriers.

### 3.2 Molecular and phylogenetic analyses

**Figure 2:**
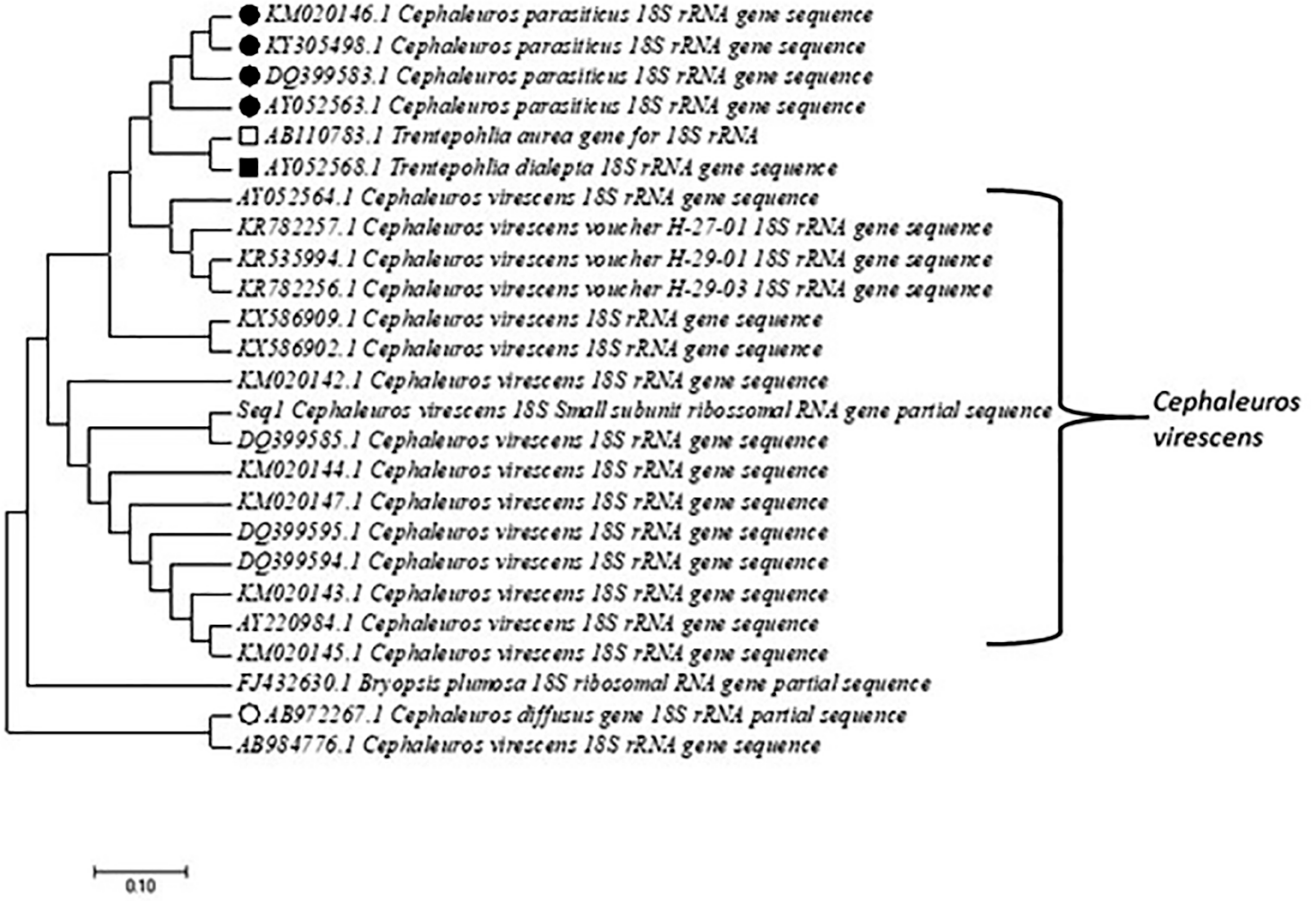
Phylogenetic tree generated from the sequencing of 18S rRNA partial gene of *C. virescens* H-27-03 (accession no. GenBank: MH042530) from African mahogany (*Khaya ivorensis*) in the Brazilian Cerrado. The phylogenetic relationships included all sequences with ≥98% identity with species of the Trentepohliaceae family, with the highest number of representatives of the genus *Cephaleuros*. Nodes reflect the consensus of maximum likelihood and Bayesian inference.

The obtained sequence was deposited in GenBank under accession number MH042530. It was subsequently compared with sequences from known species of the Trentepohliaceae family available in the National Center for Biotechnology Information (NCBI) database using the BLASTn algorithm. The alignment of our sequence (seq 1) with all the sequences deposited in GenBank that presented identity ≥98% and a coverage of 100% grouped in a single clade (major) all the species belonging to Trentepohliaceae and separated the genus *Cephaleuros* (*C. virescens* and *C parasiticus*) and *Trentepohlia* (*T. aurea* and *T. dialepta*). In particular, two species of *Cephaleuros* (*C. difusus*: AB972267.1 and *C. virescens*: AB984776.1) were outside the common group of the other species of the genus because they presented insertions/substitutions that were not observed in the other sequences assigned to these species. Both sequences showed identity ≤95% with all sequences included in the analyses presented in **Figure 2**, but we consider them important to insert in our analyses because they are reported by a respected research group with green algae of the genus *Cephaleuros*.

## 4 Discussion

### 4.1 Isolation in liquid media

The selection of the optimal medium was based on the absolute biological contrast observed, where growth was consistently absent in standard synthetic media and only achieved in specialized formulations. This deterministic approach is consistent with protocols for highly recalcitrant and slow-growing organisms, where the primary objective is the validation of a functional growth substrate rather than the measurement of quantitative growth rates. Isolation of the alga using solid media PDA, PSA, BBM, HLE, and Trebouxia from symptomatic host tissue fragments did not support algal growth. Successful biomass production required the germination of reproductive structures to initiate vegetative thallus formation. Consistent with Malagi et al. (2011), PSA medium did not enable *in vitro* growth, indicating that the alga is highly sensitive to the nutritional composition of the culture medium and requires more than a simple carbon source, as exemplified by PDA and PSA. In contrast to Suto and Ohtani (2011), who reported growth of *Cephaleuros* sp. on solid BBM medium, no algal development was observed when diseased tissue fragments were directly transferred to solid media.

*C. virescens* requires specific nutritional supplementation (Trebouxia medium) that had not been tested for this pathosystem (*C. virescens* × *K. ivorensis*). Therefore, corroboratively to Vasconcelos et al. (2018), our study suggests that several factors contributed to obtaining cellular filaments of the algae in Trebouxia liquid medium: (1) *in vitro* development of these cells requires agitation, which is achieved in liquid medium; (2) shaking at 120 rpm in liquid medium facilitates the release of sporangia from lesions, thereby enabling subsequent development of filamentous cells; (3) *Cephaleuros* sp. is considered highly sensitive to both isolation methodology and nutritional requirements; (4) commonly used solid media, such as PDA and PSA, contain only sugar and starch, which are insufficient for *in vitro* cultivation of this alga; and (5) successful cultivation is only achieved through gradual adaptation of the microorganism to artificial media. Specifically, initial isolation must occur in liquid medium, namely, Trebouxia medium, which supplies nitrogen via proteose peptone followed by transfer to solid medium after abundant filamentous cell growth. Moreover, the positive results obtained with Trebouxia medium demonstrate that peptone and infusions present in the medium serve as essential sources of carbon, nitrogen, vitamins, and amino acids for the growth of *Cephaleuros* sp (Ponmurugan et al., 2010). A static control was not included in this specific screening; the success achieved reinforces the efficacy of this physical stimulation for *Cephaleuros*.

Among the liquid media evaluated, Trebouxia medium supported greater algal growth following inoculation with surface-disinfested (D) fragments of symptomatic plant tissue, compared to non-disinfested (ND) fragments. According to Oliveira et al. (2012), surface disinfestation effectively removes contaminating microorganisms that could otherwise inhibit the growth of the target organism. Consequently, disinfestation proved beneficial for algal proliferation. In contrast to the present findings, Ponmurugan et al. (2010) reported successful cultivation of *C. parasiticus* in HLE liquid medium. The total failure of the HLE medium is noteworthy. Although phytochemical profiles of *K. ivorensis* were not obtained in this study, the observed oxidation (browning) of the medium suggests the release of inhibitory compounds, likely phenolics or tannins, as previously reported for woody hosts of *Cephaleuros* (Macêdo et al., 2020). In contrast, the richness of amino acids and vitamins in the Trebouxia medium neutralized this recalcitrance. Therefore, once growth was established in Trebouxia liquid medium, this medium was subsequently used for subculturing the alga onto solid substrate, with the aim of obtaining pure colonies of isolate H-27–03 for DNA sequencing and molecular identification.

### 4.2 Molecular and phylogenetic analyses

When identifying *Cephaleuros* at the species level, it is crucial to note that not all morphological characters are comprehensively assessed in published studies. This lack of standardization in the measurement of diagnostic traits complicates species-level identification based on micromorphological features (Vasconcelos et al., 2019).

Hamby et al. (1988) demonstrated that the 18SHf and 18SLr regions of the rRNA gene are informative markers for distinguishing pathogenic species within. Similarly, the present study confirmed the specificity of these gene regions, as their sequences remained consistent across alignments with other Trentepohliales algae, thereby validating the reliability of this molecular marker. Furthermore, species-level identification has been achieved through phylogenetic analysis based on sequencing of the 18S rRNA region, enabling direct comparison with specimens collected from tropical and subtropical environments (López-Bautista et al., 2006), which closely resemble the ecosystem in which the target alga of this study was found.

Although most of the Trentepohliales studies are focused on morphology, reproduction, distribution, and taxonomy, phylogenetic affinities, particularly the Trentepohliaceae family, still present discrepancies regarding the positioning of some divergent species (sequences), which are clearly observed for sequences deposited by different research groups around the world (Rindi et al., 2005). In this study, we present these differences (at the nucleotide level) after alignment of our sequence (sample) with all sequences deposited in GenBank that had identity ≤98% and coverage of 100%. In the evolutionary history presented in **Figure 2**, we observed that all species of the Trentepohliaceae family grouped in a single clade, except for two species of *Cephaleuros* (*C. diffusus*: AB972267.1 and *C. virescens*: AB984776.1) both described by Pitaloka et al. (2015). These authors admit the identity of 94% for both species but did not include other sequences of *C. diffusus* and *C. virescens* in their analyses, for which it would show the distance mentioned here. Further separations and categorizations of *C. virescens* species observed in **Figure 1** were caused by confounding substitutions or bases (w, k) and point deletions (gaps) but did not separate the species from the single clade. Therefore, there is no question about the grouping of these species, but we observed that a study of genetics of populations with the genus *Cephaleuros* would be quite elucidative in the phylogeny of the group, and advancement of molecular studies focused on genes of biotechnological and agronomic interest, such as the production of carotene and hematochrome, described by Rindi et al. (2009), and *algal* sp*ot* disease on higher plants caused by *C. diffusus* and *C. virescens* (Munasinghe, 1961; Marlatt and Campbell, 1980; Nelson, 2008; Visarntanon, 2010; Pitaloka et al., 2014, 2015; Sunpapao et al., 2016).

The hemiparasitic nature and extreme slow growth of *C. virescens* (often requiring months for symptom expression) impose significant barriers to the classical fulfillment of Koch’s postulates. However, the high molecular identity validation (98%) and consistent morpho-pathological association provide high diagnostic certainty (Vasconcelos et al., 2018).

None of the solid media evaluated in this study enabled the direct isolation of the green alga exhibiting algal spot symptoms on African mahogany (*K. ivorensis*) leaves from reforestation areas in the Brazilian Cerrado. In contrast, successful isolation was achieved exclusively in Trebouxia liquid medium, which supported sufficient biomass production for subsequent molecular analyses. The matrix of the aligned sequence (MH042530) generated in this work combined with partial 18S rRNA of sequences of the specimens of Trentepohliaceae family made it possible to identify our sample as *C. virescens*. Although leaf spot symptoms have been previously observed in African mahogany plantations, this work represents the first report of *C. virescens* on *K. ivorensis* in the Brazilian Cerrado supported by rigorous molecular confirmation. Previous records often relied solely on morphological features, which can be taxonomically ambiguous in the *Trentepohliales*. While the protocol’s success was demonstrated using a single isolate (H-27-03), this study establishes a robust methodological foundation. Future research involving a broader range of isolates from diverse geographic regions and host varieties will be essential to further validate the universal applicability of this liquid-to-solid transition strategy.

## Data Availability

The datasets presented in this study can be found in online repositories. The names of the repository/repositories and accession number(s) can be found below: https://www.ncbi.nlm.nih.gov/genbank/, FJ 432630.1.
